# Drug Delivery via the Suprachoroidal Space for the Treatment of Retinal Diseases

**DOI:** 10.3390/pharmaceutics13070967

**Published:** 2021-06-26

**Authors:** Liron Naftali Ben Haim, Elad Moisseiev

**Affiliations:** 1 Department of Ophthalmology, Meir Medical Center, Kfar Saba, 59 Tshernichovsky St., Kfar Saba 4428164, Israel; elad_moi@netvision.net.il; 2 The Sackler Faculty of Medicine, Tel Aviv University, Tel Aviv 6997801, Israel

**Keywords:** ocular drug delivery, ophthalmic targeting, posterior segment of the eye, suprachoroidal space, SCS, microneedle injection, gene therapy

## Abstract

The suprachoroidal space (SCS), a potential space between the sclera and choroid, is becoming an applicable method to deliver therapeutics to the back of the eye. In recent years, a vast amount of research in the field has been carried out, with new discoveries in different areas of interest, such as imaging, drug delivery methods, pharmacokinetics, pharmacotherapies in preclinical and clinical trials and advanced therapies. The SCS can be visualized via advanced techniques of optical coherence tomography (OCT) in eyes with different pathologies, and even in healthy eyes. Drugs can be delivered easily and safely via hollow microneedles fitted to the length of the approximate thickness of the sclera. SCS injections were found to reach greater baseline concentrations in the target layers compared to intravitreal (IVT) injection, while agent clearance was faster with highly aqueous soluble molecules. Clinical trials with SCS injection of triamcinolone acetonide (TA) were executed with promising findings for patients with noninfectious uveitis (NIU), NIU implicated with macular edema and diabetic macular edema (DME). Gene therapy is evolving rapidly with viral and non-viral vectors that were found to be safe and efficient in preclinical trials. Here, we review these novel different aspects and new developments in clinical treatment of the posterior segment of the eye.

## 1. Introduction

The posterior segment of the eye consists of all the structures behind the anterior hyaloid membrane, including the vitreous, retina, choroid and optic nerve [1]. The most common diseases of the posterior segment structures are age-related macular degeneration (AMD), diabetic retinopathy, diabetic macular edema, retinal vascular occlusions and noninfectious uveitis, and they are major contributors of visual impairment and blindness [2]. Current pharmacologic treatments include mainly anti-vascular endothelial growth factor (VEGF) and corticosteroids administered via intravitreal (IVT) injections which have become the first-line treatment for many retinal diseases [3].

In this review article, we discuss the suprachoroidal space (SCS) as a route of drug administration to the posterior segment of the eye. As this avenue of drug delivery is continuously evolving, our review provides comprehensive and up to date information regarding its use for treating retinal diseases. We focused mainly on the most advanced human clinical trials but also included drugs and techniques in early stages of research.

### 1.1. An Unmet Need

An ideal therapy is one that targets the exact area of pathology it is intended to treat, is easy to administer (requires little skill and can be performed as an office procedure), achieves a long-lasting effect and has a good safety profile.

**Figure 1:**
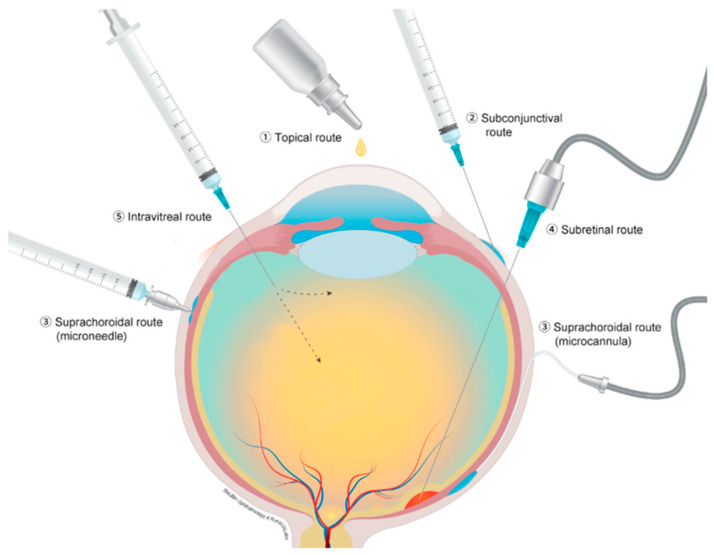
Different ocular drug administration routs; (**1**) topical route, (**2**) subconjunctival route, (**3**) suprachoroidal route with microcannula and microneedle, (**4**) subretinal route and (**5**) intravitreal injection. Reproduced from [5], CC BY 4.0 license, Published by MDPI 2021.

Current routes of drug delivery for therapeutics for conditions related to the posterior segment of the eye include topical, subtenon, subretinal and IVT injections. Figure 1 depicts different ocular drug administration routes. Each type of administration carries its own advantages and disadvantages. Topical administration is the least invasive but is often ineffective due to poor penetrance and low therapeutic levels at the posterior segment structures [4]. Subretinal injections are very targeted, yet require an invasive surgical procedure, that also carries significant risks [5]. IVT injection is easy to administer and can be performed in an office setting; however, it does not target a specific area and has adverse effects, including endophthalmitis, elevated intraocular pressure (IOP) and cataract progression [6].

Suprachoroidal (SC) injection, particularly when administered via a microneedle, carries the potential benefits of being easy to administer and better targeted to the posterior segment with fewer adverse effects, and may also allow sustained release over a long period of time. Consequently, this appealing route of administration is under extensive investigation.

### 1.2. Anatomy

The SCS is the potential space lying between the sclera and choroid [7]. The choroid and sclera are usually in close contact with each other due to the IOP [8] and attaching fibers [9], making the SCS a potential space which is created with the accumulation of fluid which may be introduced either internally or externally. In contrast to the subretinal space, the SCS is not immune privileged since it is located outside the blood–retinal barrier.

The boundaries of the SCS were found to be anterior in the scleral spur, where the sclera adheres the ciliary body, the optic nerve and the short ciliary vessels posteriorly [10,11,12,13].

### 1.3. Imaging

Until recently, the SCS was considered a theoretical space that expands in different eye pathologies, such as SC effusion and SC hemorrhage [14]. Its visualization methods included histology in ex vivo eyes [10] and ultrasonography (US) in vivo [15]. Optical coherence tomography (OCT) innovated the field with the discovery of the SCS in healthy eyes as well, enabling better visualization of the choroid in vivo. The ability to visualize the choroid with uniform interpretation is currently evolving, and in the future is expected to become an integral part of diagnosing and monitoring retinal diseases [16].

**Figure 2:**
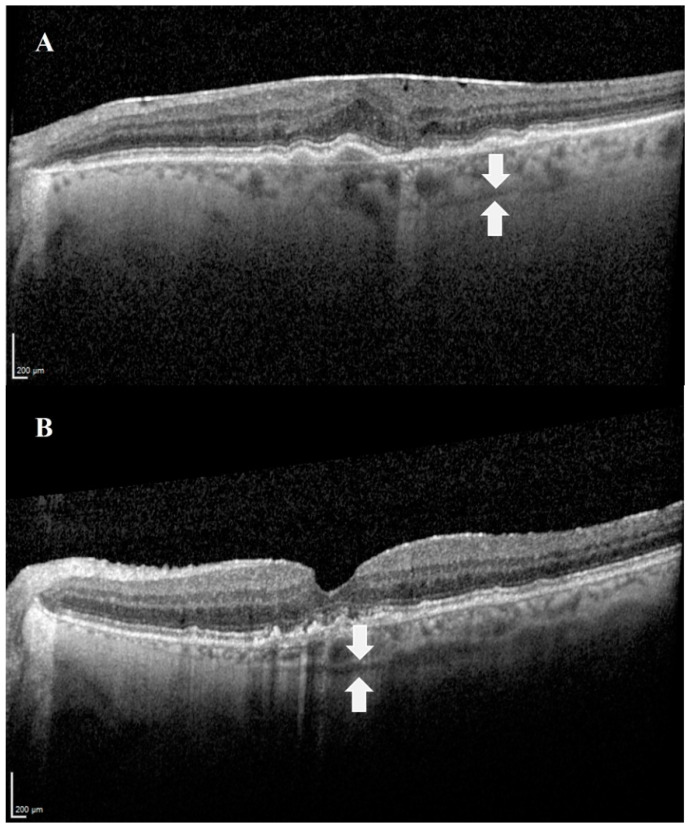
Images demonstrating the SCS in OCT. The suprachoroidal space can be visualized as a hyporeflective band (white arrows), between the choroid and the sclera. (**A**) An image of the left eye of an 80-year-old female patient with age-related macular degeneration. (**B**) An image of the left eye of a 74-year-old male patient with age-related macular degeneration.

The SCS can be visualized via advanced techniques of optical coherence tomography (OCT): swept-source OCT (SS-OCT) [17] and enhanced depth imaging OCT (EDI-OCT) [18]. EDI-OCT achieves greater depth of field by placing the OCT device closer to the eye, while SS-OCT uses a longer wavelength that allows deeper penetration. In addition, the combination of both methods, EDI SS-OCT, is optional and was found to be the most accurate modality to currently visualize the SCS in vivo compared to non-EDI SS-OCT. The combined method enables more frequent and extensive visualization of the SCS [19]. In all OCT techniques, the SCS is visualized as a hyporeflective band (Figure 2) [20].

The SCS is not visualized in all healthy patients, and is generally absent in the eyes of young, healthy persons [21,22]. The visualization is easier with increased age and is present in approximately 50% of people above the age of 50 years. Other correlations of better SCS visualization were found with hyperopia [21,23] and different pathologies such as exudative and non-exudative age-related macular degeneration (AMD) [24], macular hole, epiretinal membrane [22] and central serous chorioretinopathy (CSR) [25]. More pigmented uveal melanocytes in the choroid were found to affect SCS visibility in EDI-OCT—a darker SCS, seen in Asians and African Americans, makes it harder to visualize the SCS [26].

## 2. Drug Delivery to the SCS

### 2.1. Surgical Procedures

Surgical procedures to access the SCS have been reported in different studies [27,28,29,30,31,32]. The surgical approach includes an ab externo incision through the sclera (a.k.a. sclerotomy) and insertion of a catheter or canula in order to reach the posterior target area. These procedures carry the advantages of precise targeting and visualization since the catheter can be guided with a flashing diode [28,33]. Drawbacks include the invasiveness of the procedure, need for a skilled executor, the need of an operating room setting, risk of adverse events and complications such as SC hemorrhage, inadvertent penetration and endophthalmitis, choroidal tears, choroidal blood flow irregularities, postoperative inflammation, scleral ectasia, retinal detachment, wound abscess and others [28]. Surgical ab interno access to the SCS is currently used to insert micro stents for IOP lowering in glaucoma patients [34,35,36].

### 2.2. Standard Hypodermic Needle

Injection into the SCS can be done with the readily available standard hypodermic needle [37,38,39]. On the one hand, the material itself is available for use and the method is less invasive. On the other hand, since there is no visualization, this method requires great skill to achieve precise injection and carries the risk of inadvertently injecting into other structures, as well as causing complications such as choroidal hemorrhage and retinal detachment [40].

### 2.3. Hollow Microneedles

**Figure 3:**
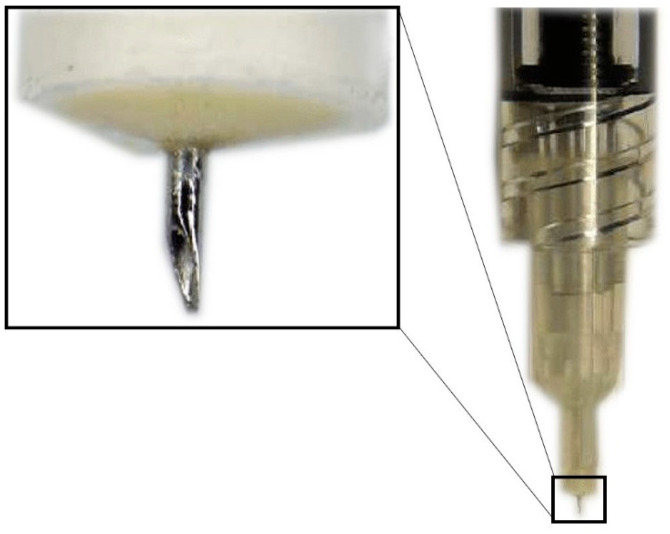
Hollow microneedle for SCS injection.

Hollow microneedles are microscopic applicators which are used most commonly to deliver drugs transdermally. They have an empty space inside which is filled with the drug dispersion or solution and have holes at the tips (Figure 3) [41]. The SCS microneedles are designed to fit the length of the approximate thickness of the sclera and carry the following advantages: they are simple to use, less painful, inexpensive, minimally invasive, require little training, can be used in an outpatient setting and have a better safety profile, making them the most promising root of administration to the SCS [42].

Patel et al. [11] showed the applicability of hollow microneedles for injecting drugs into the SCS. Their experiments were performed on rabbit, pig and human eyes ex vivo, using nanoparticle and microparticle suspensions. Their results indicate that delivery to the SCS is facilitated by increasing infusion pressure, increasing microneedle length, increasing IOP and decreasing particle size.

To date, in the setting of clinical trials, the procedure has been performed over 1000 times and was found to be reliable, consistent and with an acceptable safety profile [37,43,44,45,46,47].

## 3. Pharmacokinetics

Pharmacokinetics relate to the uptake of drugs by the body, their distribution and their elimination over a period of time [48]. In the context of SCS injection, the interest is in the distribution of the drug within the SCS (anterior vs. posterior SCS), within the eye structures (anterior and posterior segments) and within the retinal layers (retinal pigment epithelium (RPE), choroid, photoreceptors, etc.), as well as its clearance [44].

In general, SCS injections were found to reach greater baseline concentrations in the target layers—the choroid, RPE and retinal tissues compared to IVT injections [11,12,28,31,32,33,37,38,49,50]—while agent clearance was faster with highly aqueous soluble molecules [31,33,51].

### 3.1. Distribution

Material injected to the SCS was found to flow circumferentially [12,50,52] in a non-uniform pattern with the boundaries of specific anatomic structures—the scleral spur, the optic nerve and the short ciliary vessels [10,11,12,13].

The distribution seems to be related to the volume of the liquid injected and its viscosity. Studying the impact of different fluid volumes showed that increased injection volumes achieved a higher coverage area [11,12,50,52,53,54]. While Seiler et al. [55] found that the maximal SCS thickness depends on injection volumes (cannulating 250–1000 µL of 0.9% phosphate-buffered saline into porcine eyes ex vivo), Chiang et al. [53] reported SCS thickness in the area of injection to reach a maximum independent of the volume injected for low-viscosity formulations and increased SCS thickness in the area of injection, with increasing volume of high-viscosity formulations (injecting 25–150 µL of ascending viscosity solutions—Hank’s balanced salt solution (HBSS), DisCoVisc (Alcon, Fort Worth, TX, USA) and 1 to 5% carboxymethyl cellulose (CMC) in HBSS into rabbit eyes ex vivo). These results imply that low-viscosity formulations spread circumferentially, increasing the coverage area, while high-viscosity formulations stay at the area of injection, increasing the SCS thickness.

Particles, molecules and drugs injected to the SCS were found in target tissues—the choroid, RPE and retina [11,12,28,31,32,33,37,38,49,50]—while anterior segment tissues remained quite spared [12,31,33,49]. For example: Olsen et al. [33] studied 1.25 and 3 mg of bevacizumab in 0.07 mL solution in a porcine model and found that SCS bevacizumab was delivered primarily to the choroid, RPE and photoreceptor outer segments; Kim et al. [32] reported that Gd-DTPA was delivered to the posterior segment with limited anterior segment exposure after intrascleral catheterization and injection; Tyagi et al. [49] reported the highest maximum concentration of sodium fluorescein (NAF) in the choroid–retina with SCS injection in rats compared to IVT injection and posterior subconjunctival injection; and Patel et al. [12] found that the concentration of injected materials (fluorescein, fluorescently tagged dextrans, bevacizumab and polymeric particles) via SCS microneedle was at least 10-fold higher in the back of the eye tissues than in anterior tissues.

Since drugs injected into the SCS spread circumferentially, there is a need of better targeting to areas of interest. In order to better target the posterior segment and reach higher bioavailability at the sites of drug action, special formulations and techniques are being studied, including iontophoresis, swollen hydrogel pushing, high-density particles and formulations containing collagenase [56,57].

Iontophoresis, first introduced as a process of transdermal drug delivery [58], is a delivery system that utilizes an electric current as a driving force for permeation of ionic and non-ionic medications. Jung et al. [59] proposed the use of iontophoresis to direct delivery of negatively charged nanoparticles through the SCS toward the posterior pole of the eye. In their study, an electric current was applied at the conjunctiva to transfer charged molecules. They compared nanoparticle distribution with and without the use of iontophoresis in an ex vivo rabbit eye and in vivo rabbit eye. Iontophoresis using their novel microneedle-based device increased posterior pole targeting with >30% nanoparticles in the most posterior region of the SCS compared to <15% of nanoparticles without iontophoresis in the ex vivo rabbit eye. Iontophoresis in the in vivo rabbit eye resulted in approximately 30% nanoparticles delivered to the most posterior region of the SCS.

A hydrogel is a network of crosslinked polymer chains that are hydrophilic and capable of holding large amounts of water due to their structure [60]. Hyaluronic acid (HA) hydrogels are used frequently for sustained ocular drug delivery [61]. Jung et al. [62] utilized the ability of hydrogels to swell and proposed targeting the posterior SCS by using a HA hydrogel that swells and pushes the drug particles. In their study, a single syringe containing two formulations—a particle formulation and a hydrogel formulation—was used for injection into the SCS of the rabbit eye ex vivo and in vivo. Injecting only the particle formulation resulted in less than 12% of the material reaching the posterior SCS, whereas injecting the combination of formulations resulted in up to 76% of the particles being delivered to the desired area.

High-density particles injected into the SCS can target specific locations in the posterior eye, especially the macula, utilizing the force of gravity. Kim et al. [63] developed a new formulation—particle-stabilized emulsion droplets (PEDs) that consist of a perflurodecalin high-density core and stabilizing nanoparticles at the circumference. When injected into rabbit eyes oriented upward in vivo, up to 50% of nanoparticles were near the macula.

Formulations containing collagenase, an enzyme that breaks down collagen and was hypothesized to break down fibrils linking the choroid and sclera, thus impeding microparticle movement, were investigated in rabbit eyes ex vivo and in vivo. The drug coverage was expanded in both models with better results ex vivo, suggesting another method to increase posterior drug targeting [57].

### 3.2. Clearance

Visualization of the SCS after injections revealed that the SCS is expandable in a dose-dependent manner and that it can recover to pre-injection status after injected fluid is cleared [50,55]; consequently, the clearance is assessed as the time the SCS reaches a baseline level.

When IVT injection was compared to SCS injection, the aqueous soluble drugs examined were cleared significantly faster with SCS delivery [31,33,51]; examples include: bevacizumab levels that declined rapidly and were not measurable at or beyond 7 days when injected with a microcannula to the SCS compared with a gradual decline over 30–60 days with IVT injections [33]; and keratolac injected to the SCS was eliminated faster than IVT injection of keratolac with a half life of 1.19 and 3.09 h, respectively [31].

The clearance rate of different materials injected to the SCS was investigated and found to relate to the solution’s viscosity and particle size, which can both be utilized to keep material for longer periods in the SCS. Chiang et al. [53] found that higher viscosity resulted in slower SCS closure, reporting an SCS closure time of 19 min, 6 h, 2.4 days, 4.5–9.2 days with the following ascending viscosity solutions: HBSS, DisCoVisc, CMC 1% in HBSS and CMC 5% in HBSS, respectively. Particle size affects the clearance rate as well—half-lives of molecules with molecular weight from 0.3 to 250 kDa ranged from 1.2 to 7.9 h, and very small particles of 20 nm to 10 µm in size stayed in the SCS for at least 4 months [12]. A01017, a complement factor D inhibitor, is another example of small molecules’ slower clearance. When it was injected into the SCS of rabbit eyes, the half life was at least 66 days [64]. Very large macromolecules weighing 2 MDa had slower clearance as well, remaining in the SCS for 21 days [65].

In addition to viscosity and particle size, slowing the clearance can be obtained with controlled-release drug delivery systems. Different biopolymers were studied to target this issue and were found to be biocompatible and efficient, extending the lifetime of the material injected. Such biopolymers include peptide hydrogel [66], which was injected into the SCS of rabbits in vivo with a lifetime of 14.3 ± 3.3 days; poly ortho ester (POE) [67], injected into the SCS of rabbits in vivo, that was detectable for about 6 months; light-activated polycaprolactone dimethacrylate (PCM) and hydroxyethyl methacrylate (HEMA)-based gel network, entrapping bevacizumab injected into the SCS of rabbit eyes ex vivo and rat eyes in vivo, which released bevacizumab for 4 months and maintained the stability of VEGF-binding activity [38]; a surgically implanted sustained-release cyclosporine device that was effective in controlling uveitis in horses [68,69]; a sustained release formulation of dexamethasone injected to the SCS was investigated with lapotine [70] and polyurethane [71] biopolymer implants with reported half-life of 36.4 days with lapotine and dexamethasone levels for up to 42 days with polyurethane implant; and acriflavine, an inhibitor of the angiogenic factor HIF-1, incorporated to poly lactic-co-glycolic acid injected into the SCS of rats, which was found to suppress choroidal neovascularization for at least 18 weeks [72].

## 4. Pharmacotherapies in Human Clinical Trials

Pre-clinical research found triamcinolone acetonide (TA) suspension injected SC with slow clearance, high concentrations within the sclera/choroid/RPE, low exposure to the vitreous and anterior segment structures and better reduction in ocular inflammation in an acute uveitis porcine model [33,37,50,73,74]; therefore, clinical trials with SCS injection of TA (CLS-TA; Clearside Biomedical, Alpharetta, GA, USA) with or without IVT anti-VEGF agents were executed.

The clinical trials are summarized in Table 1.

### 4.1. Macular Edema Due to Retinal Vein Occlusion (ME-RVO)

#### 4.1.1. Tanzanite

A phase 2, clinical, 3 month study which enrolled 46 eyes and compared suprachoroidal injection of CLS-TA (4 mg/100 µL) in combination with IVT aflibercept (2 mg/0.05 mL) versus aflibercept (2 mg/0.05 mL) alone in patients with ME-RVO. The results indicated that combination therapy may sustain edema resolution and improve visual outcomes, and included statistically significantly reduced re-treatments of IVT injection of aflibercept (9 vs. 23 re-treatments (*p* = 0.0013) in the combination therapy vs. monotherapy, respectively), improvement from baseline in best corrected visual acuity (BCVA) letter score at months 2 and 3 (BCVA improvement of 18.9 vs. 11.3 early treatment diabetic retinopathy study (EDTRS) letters (*p* = 0.09) at month 3 in the combination and monotherapy groups, respectively) and higher percentages of edema resolution (edema resolution (CST ≤ 310 µm) was seen in 78.3 and 47.8% of patients at month 3 in the combination and monotherapy, respectively) [43].

#### 4.1.2. Sapphire

A phase 3 clinical trial that was designed to last 12 months and recruit 460 eyes with RVO. The treatment arm was treated with combination therapy of IVT aflibercept (2 mg/0.05 mL) and CLS-TA SC injections (4 mg/100 µL), while the control arm was IVT aflibercept (2 mg/0.05 mL). This study did not find any additional benefit of the combination therapy and was thus discontinued. Although it was terminated early, the reported results of 128 and 127 completed patients from the combination and control therapy, respectively, support the good safety profile of the procedure—only one case of vitreous hemorrhage and one case of retinal detachment were reported in the combined cohort [78].

#### 4.1.3. Topaz

This phase 3 clinical trial was designed similarly to the SAPPHIRE clinical trial, with the difference of using either the anti-VEGF agent aflibercept (0.5 mg/0.05 mL) or bevacizumab (1.25 mg/0.05 mL) IVT instead of aflibercept alone in both arms (treatment and control). This trial was terminated early due to the SAPPHIRE trial results [79].

### 4.2. Diabetic Macular Edema (DME)

#### 4.2.1. Hulk

A phase 1/2 clinical trial which enrolled 20 eyes with DME—10 eyes previously treated and 10 eyes treatment naïve. The treatment-naïve eyes were treated with one-time IVT aflibercept (2 mg/0.05 mL) and CLS-TA (4 mg/100 µL), while the 10 previously treated eyes were treated with CLS-TA (4 mg/100 µL) monotherapy. The reported results were a mean BCVA change of +8.5 and +1.1 EDTRS letters and mean CST decrease of 91 and 128 µm in the treatment-naïve and previously treated groups, respectively. The study demonstrated a greater benefit for treatment-naïve eyes with SC CLS-TA. Although all eyes demonstrated anatomic improvement after CLS-TA injection, mean visual improvements were minimal among previously treated patients [45].

#### 4.2.2. Tybee

A phase 2 clinical trial that enrolled 71 eyes with treatment-naïve DME. A total of 36 eyes were the active group and received CLS-TA (4 mg/100µL) and aflibercept (2 mg/0.05 mL) at baseline and week 12. The control group consisted of 35 eyes which were treated with aflibercept (2 mg/0.05 mL) at baseline, week 4, week 8 and week 12. At 24 weeks from baseline, the mean BCVA change was 11.8 and 13.8 EDTRS letters (*p* = 0.288), there was a mean CST decrease of 212.1 and 178.6 µm (*p* = 0.089) and the mean number of treatments was 2.6 and 3.6 in the active and control groups, respectively. The visual benefit was similar between the arms, with modest anatomic benefit and potential to reduce treatment burden in the active group [75].

### 4.3. Noninfectious Uveitis Macular Edema (NIU-ME)

#### 4.3.1. Peachtree

A phase 3 clinical trial enrolling 160 eyes with NIU-ME comparing SC-injected CLS-TA (4 mg/100 µL) to sham treatment at day 0 and week 12. Results at week 24 indicated 47 vs. 16% of patients gained 15 or more EDTRS letters (*p* < 0.001), the mean reduction from baseline CST was 153 vs. 18 µm (*p* < 0.001) and 13.5 vs. 72% of patients needed a rescue therapy in the treatment and control groups, respectively [46].

#### 4.3.2. Magnolia

This phase 3 clinical trial was an extension to a 48 weeks period of the PEACHTREE clinical trial which studied the injectable suspension of CLS-TA in NIU-ME. The study enrolled 33 eyes—28 CLS-TA and 5 control. The median time to rescue therapy was 257 versus 55.5 days in the treatment and control groups, respectively, and approximately 50% of treated patients did not require additional therapy up to 9 months following last CLS-TA administration [76].

### 4.4. Noninfectious Uveitis (NIU)

#### Azalea

This phase 3 clinical trial enrolled 38 eyes with NIU with or without ME for treatment with two SC injections of CLS-TA (4 mg/100 µL) at baseline and after 12 weeks. The primary objective of the study was to assess safety, which was approved; however, the efficacy parameters investigated showed improvement as well. There was an improvement in inflammation signs in most of the patients (anterior chamber (AC) cell grade 0, AC flare grade 0 and vitreous grade 0 at week 24 in 81.6, 89.5 and 89.5% of eyes, respectively), reduced need for rescue therapy (4 eyes) and modest improvement in BCVA (from 68.9 EDTRS letters at baseline to 75.9 EDTRS letters at week 24) and CST (from mean of 335.9 at baseline to 284 at week 24). The modest mean CST improvement was probably related to the NIU patients without ME [77].

### 4.5. AMD

#### 4.5.1. Dry AMD

The Limoli Retinal Restoration Technique (LLRT) autograft is being studied for dry AMD. (See advanced therapy, autograft for more information.)

#### 4.5.2. Exudative AMD

After successful preclinical studies assessing SC axitinib, a tyrosine kinase inhibitor with broad anti-VEGF properties [80], a phase 1/2a clinical trial for SCS injection of axitinib is underway [81,82].

RGX-314, an anti-VEGF protein injected as gene therapy through AAV viral vector, was found to be safe and effective in a VEGF-induced vascular leakage model in rats [83]. A phase 2 clinical trial with SC RGX-314 is currently recruiting exudative AMD patients [84].

### 4.6. Choroidal Melanoma

A phase 2 clinical trial assessing AU-011 for the treatment of choroidal melanoma is underway [85]. (See advanced therapy, viral nanoparticles bioconjugation for more information.)

## 5. Advanced Therapies

### 5.1. Gene Therapy

Gene therapy is an evolving field in general and in ophthalmology in particular. The eye is a prime target for gene therapy due to its small size, its immune privileged nature and advanced diagnostic technologies that enable its high-resolution imaging [86]. Gene therapies for inherited and non-inherited retinal diseases have been investigated in recent decades [87,88,89] via three key routes: subretinal, IVT and SC. The SC delivery form carries the advantages of being an in-office procedure and having greater posterior surface coverage, with a good penetration through the internal limiting membrane; however, there are several challenges, which include rapid clearance due to the proximity to the choriocapillaris, systemic exposure and immune response due to preexisting neutralizing antibodies, which preclude the opportunity to augment the treatment with repeated viral vector injections. Gene therapy through the SCS has undergone preclinical assessments using viral and nonviral nanoparticles.

#### 5.1.1. Viral Vector-Based Retinal Gene Delivery

Viral gene delivery utilizes the ability of a virus to inject its DNA into a host cell and to deliver the desired genetic material into the nucleus for replication [90]. Adeno-associated virus (AAV) is a DNA-based viral vector which is the most studied viral vector for SC gene therapy. AAV2, AAV5, AAV8 and AAV9 vectors [27,83,91,92,93] were studied, carrying the green fluorescent protein (GFP) in different animal models. AAV8 was also studied, carrying RGX-314, an anti-VEGF Fab fragment in a rat disease model of VEGF-induced retinal vascular leakage, and was found to be safe and effective [83].

GFP expression was observed at least in the RPE layer in all of the studies without inducing retinal detachment. The studies which compared different routes of administration [83,91] concluded that the SC approach mediates similar transduction to that seen with subretinal injection.

Ding et al. [94] elucidated the way viral vectors traverse the RPE to access photoreceptors, suggesting transcytosis, which puts them at risk of proteosomal degradation that can be suppressed via mutated, more resistant viral vectors.

Yiu et al. and Chung et al. [93,95] reported a lower systemic humoral immune response with SC AAV8, compared to IVT AAV8; however, intraocular inflammation was more evident with AAV8 delivered to the SCS, compared to the subretinal space. Furthermore, there was a decreased transgene expression after 2 and 3 months that was hypothesized to be due to cellular damage and phagocytic activity related to the increased local inflammation.

#### 5.1.2. Nonviral Gene Delivery

Nonviral gene delivery can be used for larger genes with less risk of an immune response compared to viral vector-based gene delivery [96]. Nonviral methods to deliver genes to host cells include chemical and physical methods. To date, preclinical studies have assessed the following methods of delivery to the SCS: electrotransfer (ET), liposomes and compacted DNA nanoparticles (DNPs).

In the physical method ET, the cells are briefly shocked with an electric field which is thought to create holes and increase permeability, improving gene delivery [97]. Touchard et al. [98] assessed β-galactosidase reporter gene injected to the SCS with ET and found efficient transduction of choroidal cells and RPEfor at least 1 month in adult rats. In addition, soluble vascular endothelial growth factor receptor-1 (sFlt-1) that was injected in combination with ET to a rat choroidal neovascularization (CNV) model showed promising results with a reduced CNV-induced area compared to controls.

Liposomes are vesicles formed of phospholipid bilayers which are utilized as carriers for gene therapy or drug delivery by fusing with other bilayers such as the cell membrane [99,100]. Wan et al. injected liposomes containing gene-encoding tissue inhibitor metalloproteinases-2 (TIMP-2) to the SCS in a myopia guinea pig model and found changes in collagen I and fibronectin mRNA expression over 2 weeks [101].

DNPs typically contain a polymer chain and DNA, which are oppositely charged. This special structure facilitates cellular uptake via endocytosis [102]. Kansara et al. [103] reported that in both rabbits and non-human primate models, DNPs containing luciferase injected to the SCS were well tolerated, and persistent luciferase activity was observed in both the retina and choroid. In non-human primates, luciferase activity was observed through till day 22, the last study timepoint. In rabbits, the mean luciferase activity was comparable between SC and subretinal administrations at day 7. Shen et al. [104] reported the results of SC injections of nano particles (NPs) containing different plasmids to rats. When NPs with GFP expression plasmid were injected, GFP expression was found in the photoreceptor inner and outer segments and RPE throughout the anterior retina around the entire circumference of the eye. The expression was maintained without decline for at least 8 months and multiple injections resulted in higher expression compared to a single injection. When NPs with VEGF expression plasmid were injected, subretinal neovascularization that progressed to subretinal fibrosis occurred. When NPs with VEGF-neutralizing protein, p3sFlt1Fc, were injected, they suppressed vascular leakage and neovascularization.

### 5.2. Viral Nanoparticles (VNPs) Bioconjugation

VNPs are engineered bionanomaterials utilizing the biocompatibility of viruses for the development of therapeutics, vaccines and imaging tools [105,106]. AU-011, an investigational treatment for choroidal melanoma, consists of a human papilloma virus modified to bind heparan sulphate proteoglycans that are upregulated in tumors with light activation. In a preclinical trial in a rabbit model, xenogenetic tumors were developed by human melanoma cells implanted to the SCS, which regressed after AU-011 was injected to the SCS and activated by light [107]. A phase 2 clinical trial evaluating SC administration of AU-011 in choroidal melanoma patients is underway [85].

### 5.3. Autograft

Growth factors (GF) have been found to slow retinal degeneration and cell death in animal models [108,109,110]. Limoli Retinal Restoration Technique (LLRT) autograft [111,112] consists of adipose stromal cells, platelets and adipose-derived stem cells and is aimed at achieving constant production of GF at the chorioretinal level after SC insertion. A total of 36 eyes with dry AMD were surgically implanted with LLRT autograft; the eyes were divided into two groups by the retinal thickness average (RTA) of below (group A = 14 eyes) and equal-or-above (group B = 22 eyes) 250 µm. The results indicated better visual performance in group B (with mean Logarithm of the Minimum Angle of Resolution (logMAR) BCVA change of 0.033 and 0.18 in group A and B after 6 months, respectively (*p* = 0.04)) [111]. A subsequent study demonstrated a similar trend with improved mean logMAR BCVA, from 0.581 at baseline to 0.376 at 180 days (*p* < 0.01) in 11 grafted eyes with dry AMD [112].

### 5.4. Mesenchymal Stem Cells

Allogenic tissue-derived mesenchymal stem cells were surgically implanted into the SCS of patients with advanced diseases as a rescue therapy. Umbilical cord- [113] and adipose tissue [114]-derived mesenchymal stem cells were surgically implanted in patients with optic atrophy. Adipose tissue-derived mesenchymal stem cells were surgically implanted to the SCS of patients with dry AMD and Stargardt’s macular dystrophy [115]. All of these small studies resulted in encouraging results with BCVA and visual field improvements.

## 6. Conclusions

The SCS is a promising route to administer drugs and advanced therapies to treat posterior segment diseases. In recent years, the delivery method was well developed with minimally invasive microneedle injections, more is known about the distribution and clearance of different particles and formulations, better targeting and sustained release formulations are being investigated, clinical trials found CLS-TA injected to the SCS to be safe and effective for the treatment of NIU, NIU-ME and DME and gene therapy with viral and non-viral vectors was found to be possible and successful in preclinical trials.

Yet, challenges and open issues remain and necessitate more research in the field. First, the fast clearance of aqueous soluble particles via the SCS deserves the invention and examination of new formulations that extend the time periods in order to enable longer intervals of drug administration; second, not all the material injected to the SCS near the limbus flows to the posterior parts—more studies on better targeting with SCS injection are needed; third, clinical trials included small groups, and more clinical trials with larger groups are needed; fourth, the TANZANITE clinical trial for treatment of CLS-TA in combination with IVT aflibercept found promising results that were not repeated by subsequent clinical trials, so it might be relevant to study the role of SC CLS-TA in RVO again; fifth, the current clinical trials were performed with only one agent injected into the SCS—CLS-TA, since it has a favorable distribution and clearance profile—and although bevacizumab was found to be cleared fast from the SCS in preclinical studies, more has to be done in order to find ways to inject anti-VEGF therapies to the SCS efficiently; and sixth, there is plenty to discover about the efficacy, safety, biodegradability, immunity and inflammatory characters of advanced therapy methods for better understanding and applicability of clinical trials.

In conclusion, despite the challenges, which will be addressed in future studies, it seems that the SCS is a promising route of drug delivery that will likely become an integral part of the treatment of retinal diseases.

## Figures and Tables

**Table 1:** SCS pharmacotherapies in human clinical trials.

Clinical Trial	Study Focus	Disease	Eyes (N)	Drug	Main Results	Number of Treatments	Adverse Events	Key Points	Reference
TANZANITE (NCT02303184)	Suprachoroidal triamcinolone acetonide for retinal vein occlusion: results of the Tanzanite study	ME-RVO	Combination arm = 23 eyes	CLS-TA (4 mg/100 µL) in combination with IVT aflibercept (2 mg/0.05 mL) followed by monthly intravitreal aflibercept injections as needed	Mean BCVA improvement of 18.9 EDTRS letters at month 3 Mean CST from 731.1 µm at baseline to 285.4 µm at 4 months	9 retreatments. 78% of patients with no retreatments	1 eye cataract progression 2 eyes IOP elevation 8 patients reported eye pain	Combination therapy was well tolerated and reduced additional IVT aflibercept injections	[43]
			Monotherapy arm = 23 eyes	Aflibercept (2 mg/0.05 mL) followed by monthly intravitreal aflibercept injections as needed	Mean BCVA improvement of 11.3 EDTRS letters at month 3 (*p* = 0.09) Mean CST of 727.5 µm at baseline to 384.6 µm at 3 months	23 retreatments (*p* = 0.013), 30% of patients with no retreatments (*p* = 0.003)	No cataract and IOP elevation 1 eye with reported eye pain		
HULK (NCT02949024)	Suprachoroidal space alterations following delivery of triamcinolone acetonide: post hoc analysis of the phase 1/2 HULK study of patients with diabetic macular edema	DME	Treatment-naïve group = 10 eyes	1-time IVT aflibercept (2 mg/0.05 mL) and CLS-TA SC (4 mg/100 µL)	At 6 months Mean BCVA change of +8.5 EDTRS letters Mean CST reduction of 91 µm	Mean of 2.6 CLS-TA injections	2 patients with IOP rise, 3 cases of cataract progression, 1 case of local pain during SC injection, 1 case of inadvertent IVT injection	Anatomic improvements in all eyes after CLS-TA injection. SC injection of CLS-TA was safe in DME population. Greater benefit for treatment naïve eyes with SC injection of CLS-TA.	[45]
			Previously treated group = 10 eyes	CLS-TA SC (4 mg/100 µL) monotherapy	At 6 months Mean BCVA change of +1.1 EDTRS letters Mean CST reduction of 128 µm	Mean of 3.3 CLS-TA injections			
TYBEE (NCT03126786)	Suprachoroidal CLS-TA plus intravitreal aflibercept for diabetic macular edema: a randomized, double-masked, parallel-design, controlled study	DME	Active group = 36 eyes	CLS-TA (4 mg/100 µL) and aflibercept (2 mg/0.05 mL) at baseline and week 12	At 24 weeks Mean BCVA change of 11.8 EDTRS letters Mean CST decrease of 212.1µm	Average of 2.6 treatments (baseline CLS-TA and then aflibercept)	Increased IOP—3 patients Cataract—2 patients	The visual benefit was similar between the arms. SC CLS-TA may address treatment burden. SC CLS-TA is safe, with no SAE, and with no difference in AE between the arms.	[75]
			Control group = 35 eyes	Aflibercept (2 mg/0.05 mL) at baseline, week 4, week 8 and week 12	At 24 weeks Mean BCVA change of 13.8 EDTRS letters (*p* = 0.288) Mean CST decrease of 178.6 µm (*p* = 0.089)	Average of 4.6 treatments (aflibercept only)	Increased IOP (>10 from baseline)—1 patient Cataract—1 patients		
PEACHTREE (NCT02595398)	Efficacy and safety of suprachoroidal CLS-TA for macular edema secondary to noninfectious uveitis: Phase 3 randomized trial	NIU-ME	Treatment group = 96 eyes	CLS-TA (4 mg/100 µL) at day 0 and week 12	47% of patients gained 15 or more ETDRS letters at week 24 Mean reduction in CST from baseline of 153 µm at week 24	13.5% of patients needed a rescue therapy	Elevated intraocular pressure occurred in 11.5% Cataract rates—7.3% Eye pain—12.5%	Clinically meaningful improvements in vision for nearly half of the patients treated.	[46]
			Sham treatment = 64 eyes	Sham procedure at day 0 and week 12	16% of patients gained 15 or more ETDRS letters at week 24 Mean reduction in CST from baseline of 18 µm at week 24	72% of patients needed a rescue therapy	Elevated intraocular pressure occurred in 15.6% Cataract rates—6.3%Eye pain 4.7%		
MAGNOLIA (NCT02952001)	Extension study of the safety and efficacy of CLS-TA for treatment of macular edema associated with noninfectious uveitis (MAGNOLIA)	NIU-ME	Treatment group = 28 eyes	CLS-TA (4 mg/100 µL) at day 0 and week 12	From CLS-TA patients not requiring rescue: Mean gain in BCVA of 12.1 EDTRS letters at week 48 Mean CST reduction of 174.5 µm at week 48	11 patients (39.3%) received rescue treatment Median time to rescue therapy was 257 days Of 28 CLS-TA treated patients who participated in MAGNOLIA, 14 (50%) did not require rescue therapy for approximately 9 months after the second treatment	4 patients (14.3%) had an IOP elevation 7 patients (25%) with cataract	Approximately 50% of patients did not require additional treatment for up to 9 months following the last CLS-TA administration	[76]
			Sham treatment = 5 eyes	Sham procedure at day 0 and week 12		3 patients (60%) received rescue treatment Median time to rescue therapy was 55.5 days	No patients with IOP elevation 1 patient (20%) with cataract		
AZALEA (NCT03097315)	Suprachoroidal CLS-TA for noninfectious uveitis: an open-label safety trial (AZALEA)	NIU	38 eyes 20 NIU-ME 18 NIU	Two SC injections of CLS-TA (4 mg/100 µL) at baseline and after 12 weeks	AC cells grade 0 from 44.7% of eyes at baseline to 81.6% of eyes at week 24 AC flare grade 0 from 71.1% of eyes at baseline to 89.5% of eyes at week 24 Vitreous haze grade 0 from 44.7% of eyes at baseline to 89.5% of eyes at week 24 Mean BCVA at baseline of 68.9 EDTRS letters improved to 75.9 EDTRS letters by week 24 Mean CST of 335.9 µm at baseline improved to 284 µm by week 24	4 eyes received rescue therapy	6 patients had IOP rise (>10 from baseline) 4 patients—cataract formation 3 patients—eye pain during SC injection	SC injection of CLS-TA was safe and well tolerated. Efficacy parameters showed improvement over 24 weeks (improvement in signs of inflammation and the need of rescue therapy).	[77]

## Data Availability

Not applicable.
